# Changes in the Distribution of Periodontal Nerve Fibers during Dentition Transition in the Cat

**DOI:** 10.1371/journal.pone.0129826

**Published:** 2015-06-17

**Authors:** Koji Miki, Shiho Honma, Satomi Ebara, Kenzo Kumamoto, Shinya Murakami, Satoshi Wakisaka

**Affiliations:** 1 Department of Oral Anatomy and Developmental Biology, Osaka University Graduate School of Dentistry, Osaka, Japan; 2 Department of Periodontology, Osaka University Graduate School of Dentistry, Osaka, Japan; 3 Department of Oral Health Sciences, Faculty of Nursing and Health Care, Baika Women’s University, Osaka, Japan; 4 Department of Anatomy, Meiji University of Integrative Medicine, Kyoto, Japan; Glasgow University, UNITED KINGDOM

## Abstract

The periodontal ligament has a rich sensory nerve supply which originates from the trigeminal ganglion and trigeminal mesencephalic nucleus. Although various types of mechanoreceptors have been reported in the periodontal ligament, the Ruffini ending is an essential one. It is unknown whether the distribution of periodontal nerve fibers in deciduous teeth is identical to that in permanent teeth or not. Moreover, morphological changes in the distribution of periodontal nerve fibers during resorption of deciduous teeth and eruption of successional permanent teeth in diphyodont animals have not been reported in detail. Therefore, in this study, we examined changes in the distribution of periodontal nerve fibers in the cat during changes in dentition (i.e., deciduous, mixed and permanent dentition) by immunohistochemistry of protein gene product 9.5. During deciduous dentition, periodontal nerve fibers were concentrated at the apical portion, and sparsely distributed in the periodontal ligament of deciduous molars. During mixed dentition, the periodontal nerve fibers of deciduous molars showed degenerative profiles during resorption. In permanent dentition, the periodontal nerve fibers of permanent premolars, the successors of deciduous molars, increased in number. Similar to permanent premolars, the periodontal nerve fibers of permanent molars, having no predecessors, increased in number, and were densely present in the apical portion. The present results indicate that the distribution of periodontal nerve fibers in deciduous dentition is almost identical to that in permanent dentition although the number of periodontal nerve fibers in deciduous dentition was low. The sparse distribution of periodontal nerve fibers in deciduous dentition agrees with clinical evidence that children are less sensitive to tooth stimulation than adults.

## Introduction

The periodontal ligament receives dense sensory innervation by nociceptive-free nerve endings and mechanoreceptive specialized endings. Although various types of mechanoreceptors have been reported in the periodontal ligament, the Ruffini ending is an essential one. The morphology and histochemical properties of periodontal Ruffini endings have been extensively studied in the periodontal ligament of rat incisors under normal conditions, and those during development and regeneration after nerve injury. Under normal conditions, periodontal Ruffini endings of the rat incisor are concentrated in the alveolar half of the lingual periodontal ligament, and display dendritic ramifications with expanded axon terminals through an association with specialized Schwann cells called terminal or lamellar Schwann cells [1, 2, 3]. Although the rodent incisors are the most popular experimental model for the examination of periodontal innervation, they have unique characteristics, i.e., they are rootless and continuously erupting. In this regard, the rat incisor is quite different from the molars. In rat molars, the Ruffini endings are mostly localized in the apical region, and have a similar morphology to the rat incisor [4, 5, 6].

One of the most apparent differences between human and rat or mouse dentition is that rat and mouse are monophyodonty, while human is diphyodonty. Thus, it is impossible to examine changes in the distribution of periodontal nerve fibers during transition of dentitions in monophyodont animals. Although the morphological studies of periodontal nerve fibers have revealed that periodontal nerve fibers were densely present in the apical portion of the periodontal ligament in permanent teeth of diphyodont animals, such as the cat [5, 7, 8, 9, 10, 11], dog [12], monkey [5, 13] and human [6, 14], it is unknown whether distribution of periodontal nerve fibers in deciduous teeth was identical to that in permanent teeth or not. Moreover, morphological changes in the distribution of periodontal nerve fibers during resorption of deciduous teeth and eruption of successional permanent teeth in diphyodont animals have not been reported in detail. Therefore, in this study, we used immunohistochemistry for protein gene product 9.5 (PGP 9.5), a pan-neuronal marker, to reveal changes in the distribution of periodontal nerve fibers of the premolar and molar teeth during the transition from deciduous to permanent dentition.

## Materials and Methods

### Animals

In the present study, cats aged 2 (n = 2), 5 (n = 2) and 12 months (n = 2) were used. The date of birth was confirmed at Meiji University of Integrative Medicine, where they were bred. This study was carried out in strict accordance with the recommendations in the Guide for the Care and Use of Laboratory Animals. The experimental protocol was reviewed and approved by the Intramural Animal Use and Care Committee of both Meiji University of Integrative Medicine (Permit Number: 22–30) and Osaka University Graduate School of Dentistry (Permit Number: 19-025-0), prior to the commencement of experiments. All surgery was performed under sodium pentobarbital anesthesia, and all efforts were made to minimize suffering.

### Tissue Preparation

Animals were paralyzed by intramuscular injection of ketamine hydrochloride (15 mg/kg) and then deeply anesthetized by intravenous injection of sodium pentobarbiturate (30 mg/kg). They were perfused transcardially with 0.02 M phosphate-buffered saline (PBS; pH 7.4) followed by 4% paraformaldehyde in 0.1 M phosphate buffer (PB; pH 7.2). Mandibles were removed and further fixed in 4% paraformaldehyde in 0.1 M PB for 2–3 days, and were examined by radiography to confirm the developmental stage of dentition. Specimens were decalcified with 7.5% ethylenediaminetetraacetic acid (EDTA) for 2–4 months at 4°C under gentle agitation. The decalcifying solution was changed at least twice a week. The decalcified tissue blocks were soaked in 20% sucrose/PBS for cryoprotection, sectioned at a thickness of 40 μm with a cryostat, collected in PBS and treated as floating sections.

### Immunohistochemistry

Sections were treated with PBS containing 0.03% H_2_O_2_ to inactivate endogenous peroxidase activity for 30 min at room temperature. Following preincubation with PBS containing 1% normal swine serum (Dako, Copenhagen, Denmark), 1% normal goat serum (Vector Laboratories, Burlingame, CA, USA) and 1% bovine serum albumin (Sigma, St. Louis, MO, USA) for 30 min, sections were incubated either with rabbit polyclonal anti-PGP9.5 (1:5000, Ultraclone, Cambridge, UK, #RA-95101) or with rabbit polyclonal anti-S100 (1:5000; Dako, #Z0311) for 16–18 h at room temperature. Following several rinses in PBS, sections were incubated with biotinylated swine anti-rabbit IgG (1:500; Dako, #E0353), and subsequently incubated with ABC complex (Vector Laboratories, Burlingame, CA, USA). Horseradish peroxidase activity was visualized by incubation with 0.05 M Tris-HCl buffer (pH 7.5) containing 0.08% diaminobenzidine (DAB) and 0.003% H_2_O_2_ enhanced with 0.1% nickel ammonium sulfate. Following the DAB reaction, immunostained sections were mounted on gelatin-subbed glass slides and lightly counterstained with methyl green. Sections were dehydrated through an ascending series of ethanol, cleared with xylene and cover-slipped with Permount (Fisher Scientific Inc., NJ, USA).

For double-labeling, sections were incubated either with a mixture of guinea pig monoclonal anti-PGP9.5 (1:1000; Chemicon, Temecula, CA, USA, #AB5898) and rabbit polyclonal anti-S100 protein (1:1000; Dako, #Z0311), or a mixture of rabbit polyclonal anti-PGP9.5 (1:5000, Ultraclone, #RA-95101) and mouse monoclonal anti-S100ß (1: 1000; Sigma, #S2532) for 16–18 h at room temperature. Following several rinses, sections were then incubated either with a mixture of Alexa Fluor 488-conjugated goat anti-rabbit IgG (1:500; Molecular Probes, Eugene, OR, #A11008) and Cy3-conjugated goat anti-mouse IgG (1:500; Jackson Immuno Research Laboratories Inc., West Grove, PA, #715-166-151), or Cy3-conjugated goat anti-guinea pig IgG (1:500; Jackson Immuno Research Laboratories Inc., West Grove, PA, #106-165-003) The immunostained sections were cover-slipped with Vectorshield (Vector) and observed under a fluorescent microscope (AxioSkop 2; Carl Zeiss, Jena, Germany). The images were captured with CCD camera software (AxioCom Ver. 4.6; Carl Zeiss), and transferred to Adobe Photoshop (CS6).

Immunohistochemical controls were performed by omission of primary antibody, biotinylated secondary antibody or ABC complex, and those sections did not show any immunoreactions.

### Quantitative Analysis

**Fig 1:**
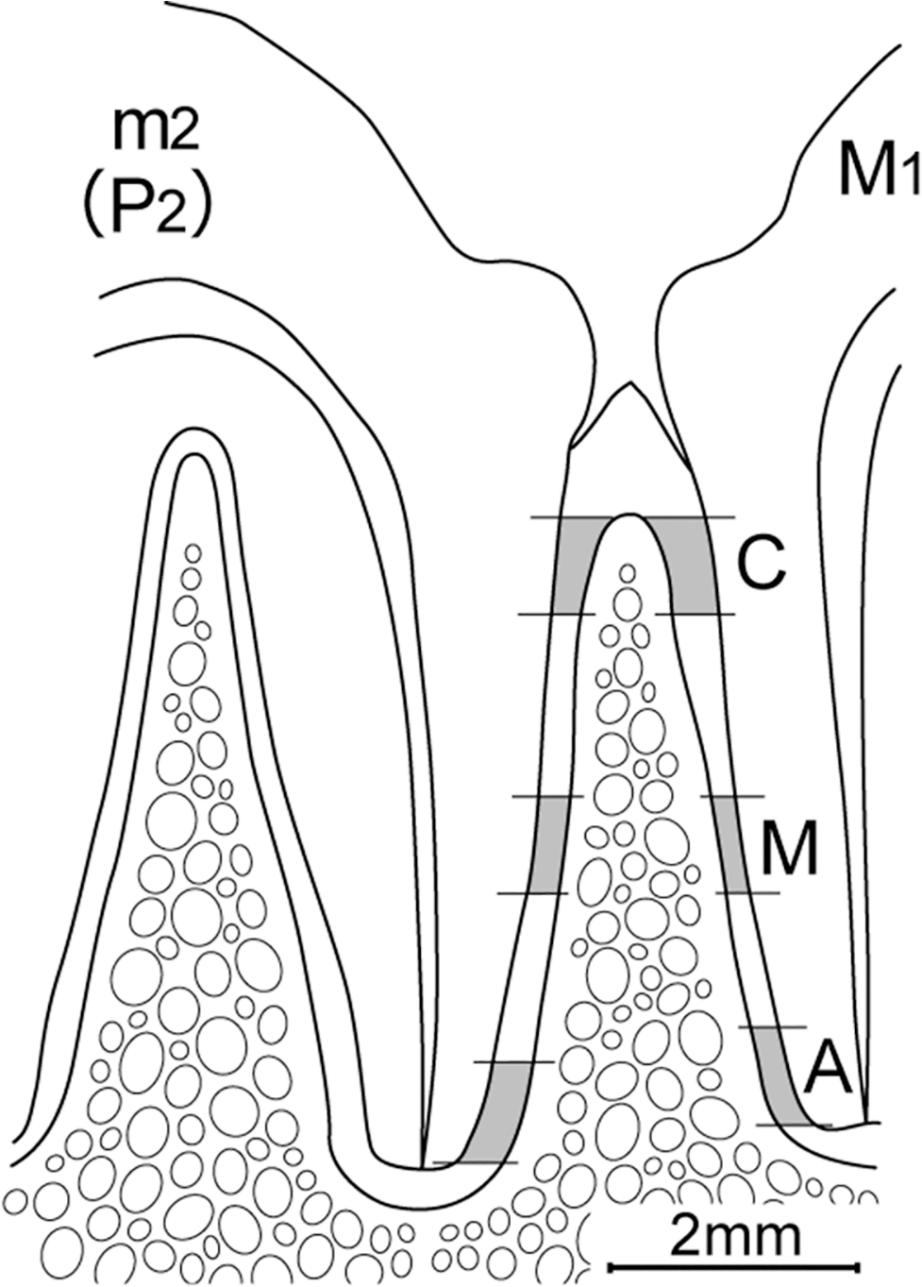
Schematic drawing for quantitative analysis. The percentage of PGP 9.5 immunoreactive areas was measured in the cervical (C), middle (M) and apical (A) portions of distal side of distal root of deciduous second molar (m_2_) of the 2-month-old cat and permanent second premolar (P_2_) of the 5-month-old cat, and mesial side of mesial root of permanent first molar (M_1_) of the 5- and 12-month-old cats at the length of 800 μm (dotted area).

For quantitative analyses, the percentages of immunoreactive areas at the distal sides of the distal roots of the deciduous second molar in the 2-month-old cats and permanent second premolar, a successor of the deciduous second molar, in the 12-month-old cats, and the mesial sides of the mesial roots of the permanent first molar in the 5- and 12-month-old cats were measured in four randomly-selected sections containing the apical foramen (two sections from each animal separated by at least 80 μm) according to our previous protocol [15], except we used Image J software package (ver. 1.47; National Institutes of Health, Bethesda, MD) for the present measurements. The measurements were made in areas at the cervical, middle, and apical portions of the periodontal ligament with 800 μm in length (Fig 1). The total areas and immunoreactive areas were measured, and the percentages of the immunoreactive areas to the total areas were calculated. The statistical significance of differences was assessed by an unpaired Student's *t*-test for comparisons between the percentages of immunoreactive areas of the deciduous second molar of the 2-month-cats and the permanent second premolar of the 12-month-old cats, and between the permanent first molar of the 5- and 12-month-old cats. Values of P < 0.05 were considered to indicate significant differences.

## Results

Dental formula of cat deciduous dentition and permanent dentition are i3/3 c1/1 m3/2 = 26 and I3/3 C1/1 P3/2 M1/1 = 30, respectively. In the present study, we focused on the distribution of the periodontal ligament of the lower deciduous second molar and its successor, the lower permanent second premolar. We also examined the distribution of periodontal nerve fibers in the lower permanent first molar, which has no predecessor tooth.

### Radiography

**Fig 2:**
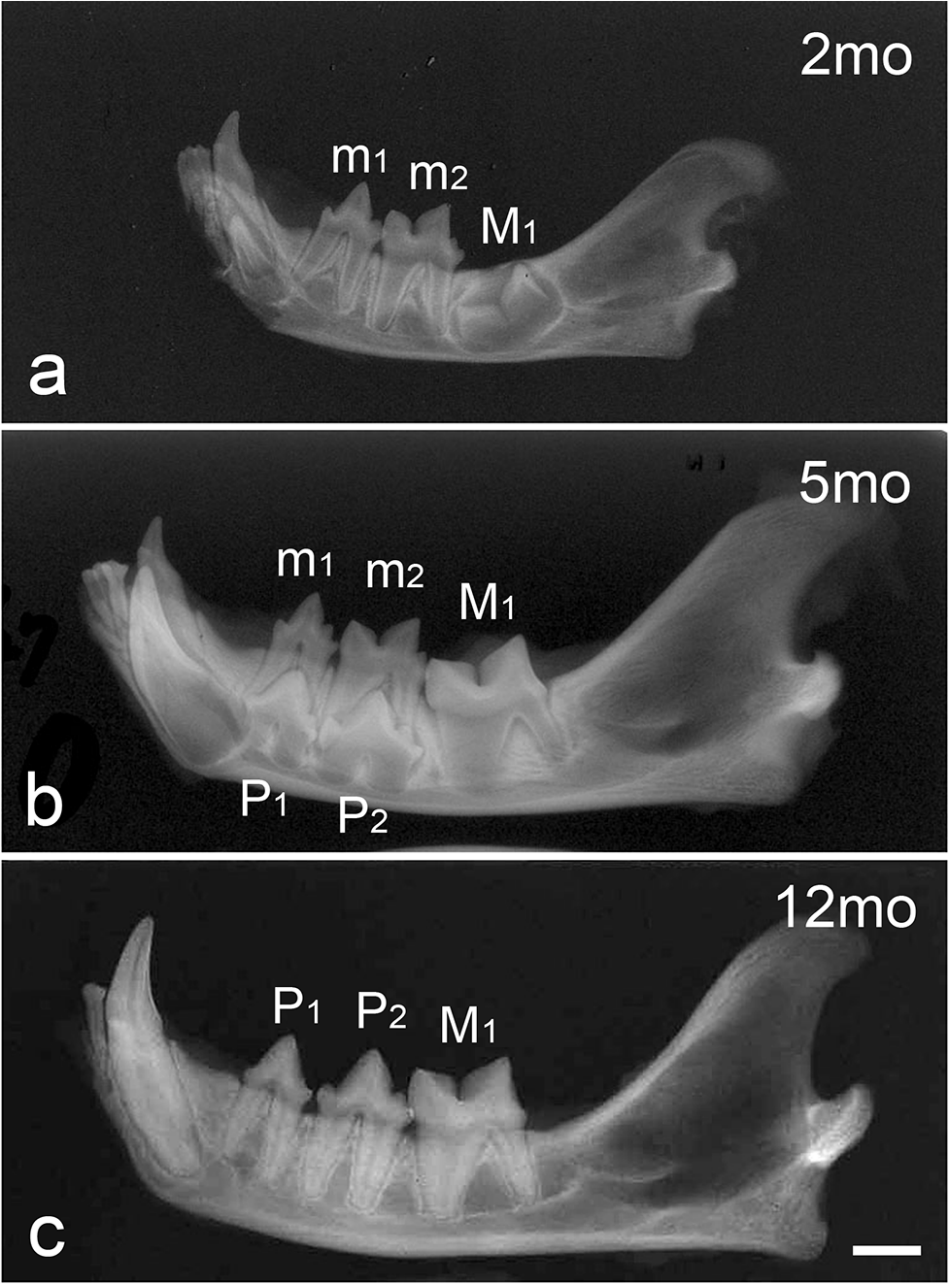
Radiographic images of the mandibles in 2-month-old (a), 5-month-old (b), and 12-month-old (c) cats. m1: first deciduous molar, m2: second deciduous molar, M1: first permanent molar, P1: first permanent premolar, P2: second permanent premolar. Scale bar = 5 mm

Radiography showed that root formation in the deciduous molars was complete in the 2-month-old cat. Root resorption had not yet begun, and the crown of the permanent first molar was almost formed (Fig 2A). In the 5-month-old cat, the developing permanent premolars induced root resorption of the deciduous molars after they began to erupt. The root formation of permanent first molar was in progress, but not completed (Fig 2B). In the 12-month-old cat, all deciduous teeth had shed, and all permanent teeth had erupted. The root formation of permanent teeth was also complete (Fig 2C).

### Immunohistochemistry

**Fig 3:**
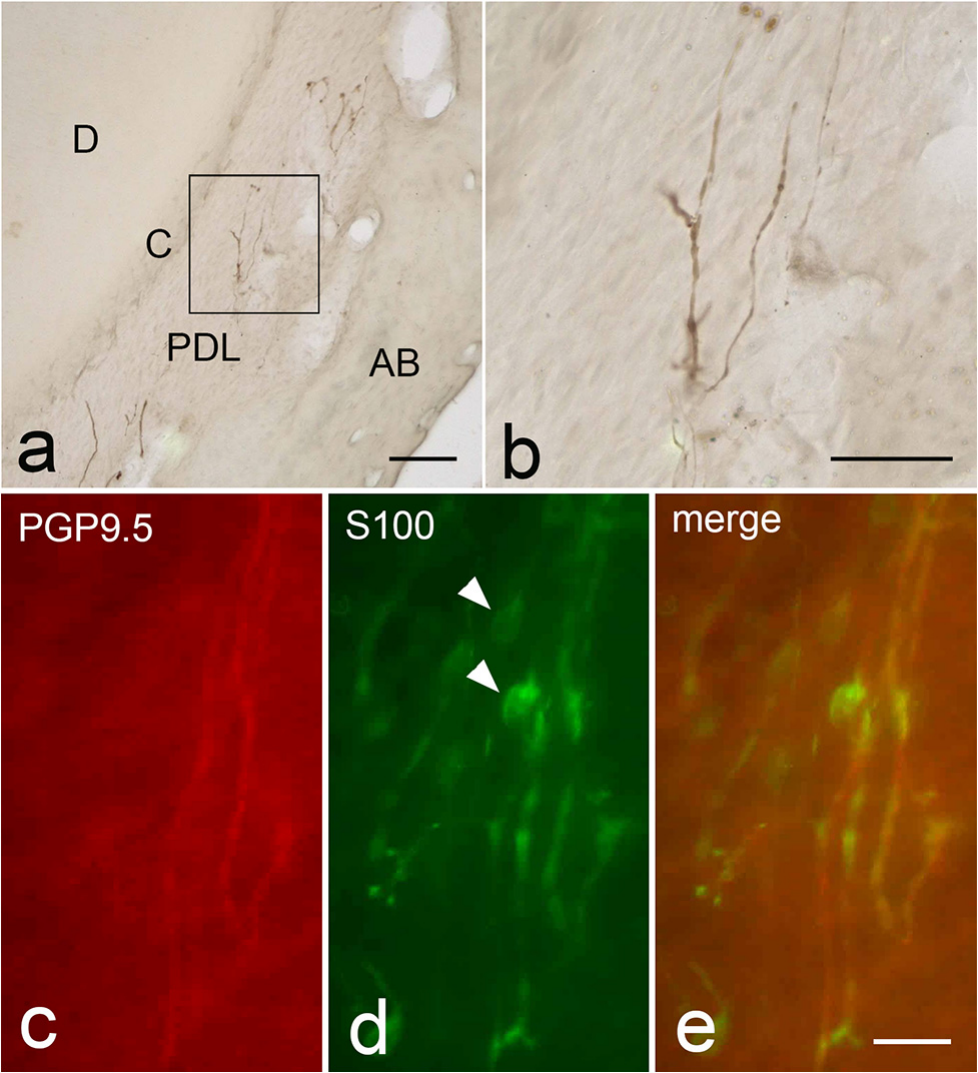
PGP9.5-immunoreactivities in the periodontal ligament of the lower second deciduous molar of the 2-month-old cat. a; Low-powered image of the root apex. A few nerve fibers are found in the periodontal ligament (PDL). C; cementum, AB; alveolar bone. b; High-powered image of the boxed area in a. Thick nerve fibers run along the periodontal ligament. They are less ramified and do not terminate in the cementum. c-e: Double-labeling for PGP9.5 (c) and S100 (d), and merged image (e) in the periodontal ligament in a 2-month-old lower second deciduous molar. Rounded S100-positive cells (arrowheads in d, e) are associated with PGP9.5-immunoreactive nerve fibers. Scale bars: 100 μm in a, 50 μm in b, 20 μm in e (also applied for c and d)

In the 2-month-old cat, thick PGP9.5-immunoreactive nerve fibers were detected in the periodontal ligament around the root apex of the deciduous molar (Fig 3A and 3B). The nerve fibers were thick, slightly ramified, and they did not penetrate into the cementum. Thin nerve fibers were rarely found. Double-fluorescent images revealed that some S100-positive round cells were found among PGP9.5-positive thick nerve fibers (Fig 3C and 3D).

**Fig 4:**
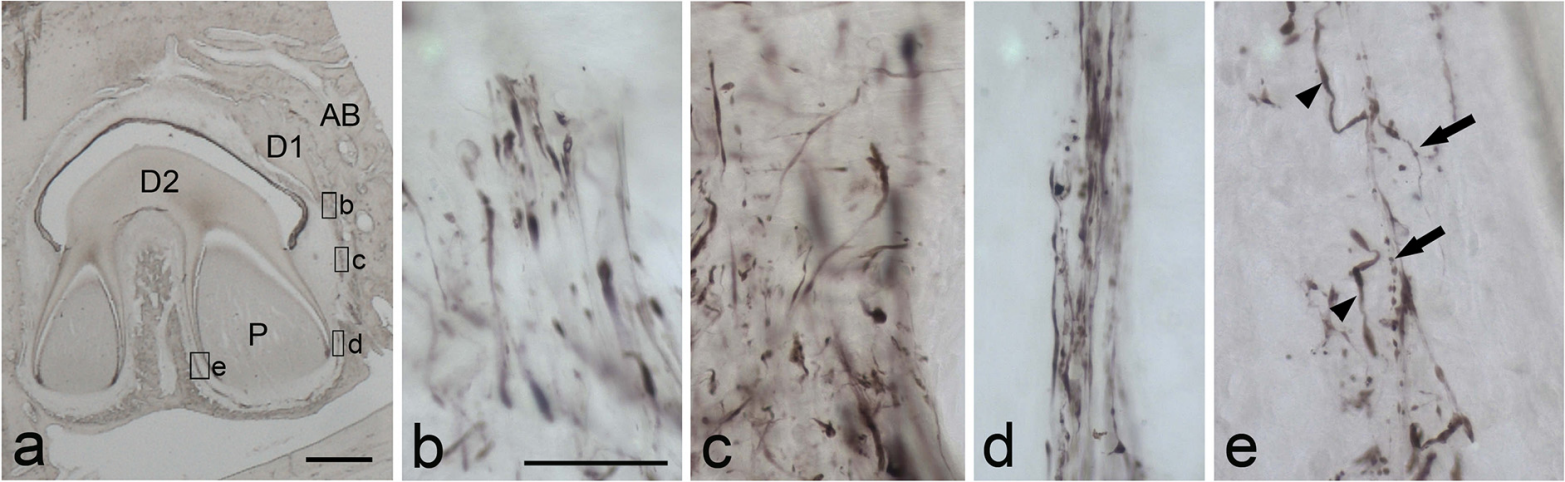
PGP9.5-immunoreactive nerve fibers in a deciduous second molar and succeeding permanent second molar in a 5-month-old cat (a). b-e: Higher magnification views of boxed areas in a. b: In the periodontal ligament under the resorbing root of deciduous second molar, PGP9.5-immunoreactive thick nerve fibers are detected as having a dot-like appearance. c: Many PGP9.5-immunoreactive nerve fibers are randomly distributed in the cervical area of the periodontal ligament of the permanent premolar. d: At the apical part of the distal side of the periodontal ligament of the distal root of the permanent second premolar, thick nerve fibers and thin varicose nerve fibers are running coronally. e: In the furcation area of the permanent premolar, PGP9.5-immunopositive thick nerve fibers (arrowheads) and thin nerve fibers with varicosity (arrows) are found. AB: alveolar bone, D1: dentin of primary teeth, D2: dentin in permanent teeth, P: pulp. Scale bars: 200 μm in a, and 50 μm in b, which is applicable to c-e.

**Fig 5:**
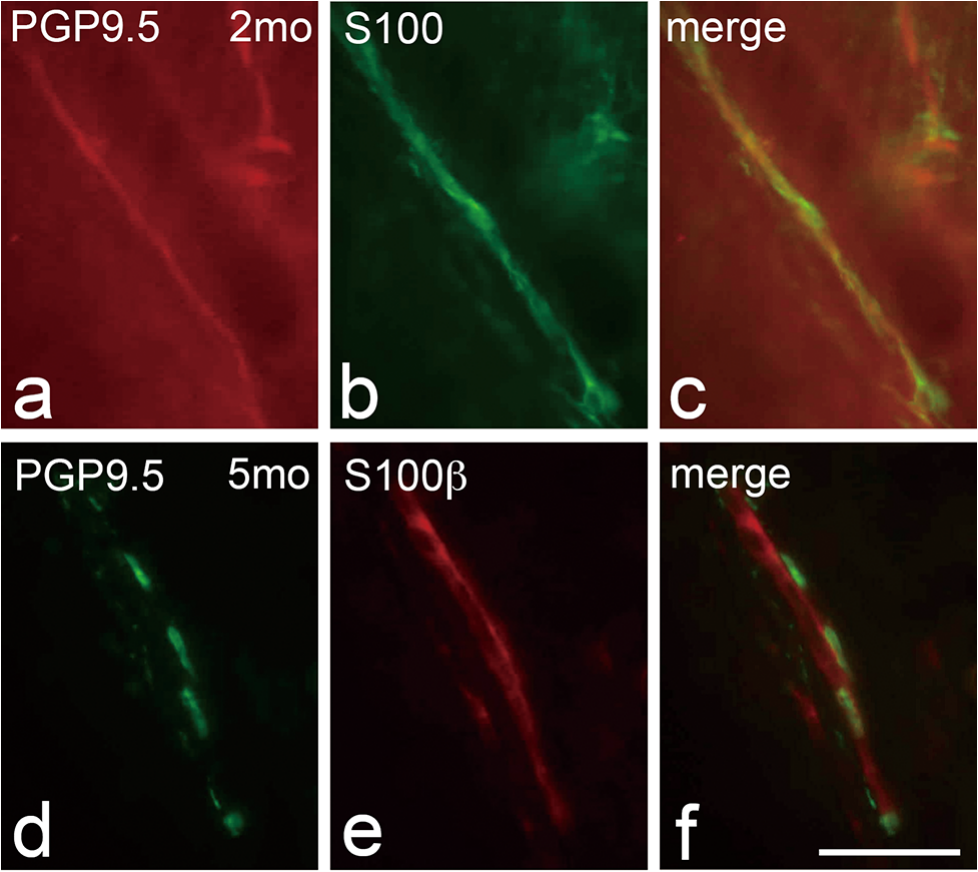
Double-labeling images for PGP9.5 (a, d) and S100 (b) or S100β(e), and merged images (c, f) in the apical periodontal ligament of deciduous second molar of 2-month-old cat (2mo; a-c) and cervical portion of erupting permanent second premolar of 5-month-old cat (5mo; d-f). a-c: PGP 9.5-immunoreactive nerve fibers (a, c) accompanying S-100 immunoreactive structures (b, c) are observed without interruption in the ligament of 2-month-old-cat. d-f: PGP9.5-immunoreactive nerve fibers show a dot-like appearance (d, f), while continuity of S100 structures is recognized (e, f) in 5-month-old cat. Scale bar: 50 μm.

In the 5-month-old cat, the root of the deciduous molar was undergoing resorption and the permanent premolars were succeeding under the deciduous molars (Fig 4A). Many thick PGP9.5-immunoreactive nerve fibers were detected as dot-like structures at the root of the resorbing area of the deciduous molar (Fig 4B) and near the coronal and cervical parts of the root of the permanent premolar (Fig 4C). PGP9.5-immunoreactive nerve fibers grew coronally at the apical portion of the permanent premolar, with some having a dot-like appearance (Fig 4D). At the furcation area of the succeeding permanent premolar, thin nerve fibers with beaded appearance and thick nerve fibers were observed (Fig 4E). Although double-fluorescent images in the periodontal ligament of deciduous second molar of 2-month-old cat showed the PGP 9,5-immunoreactive nerve fibers accompanying S-100 immunoreactivity without interruption (Fig 5A–5C), periodontal ligament of the coronal side of the erupting second permanent premolar in 5-month-old cat showed that PGP9.5-immunoreactive nerve fibers were also accompanied by S100β-immunoreactivity; PGP9.5-immunoreactive fibers showed a dot-like appearance, while S100β-immunoreactivity was homogenous (Fig 5D–5F).

**Fig 6:**
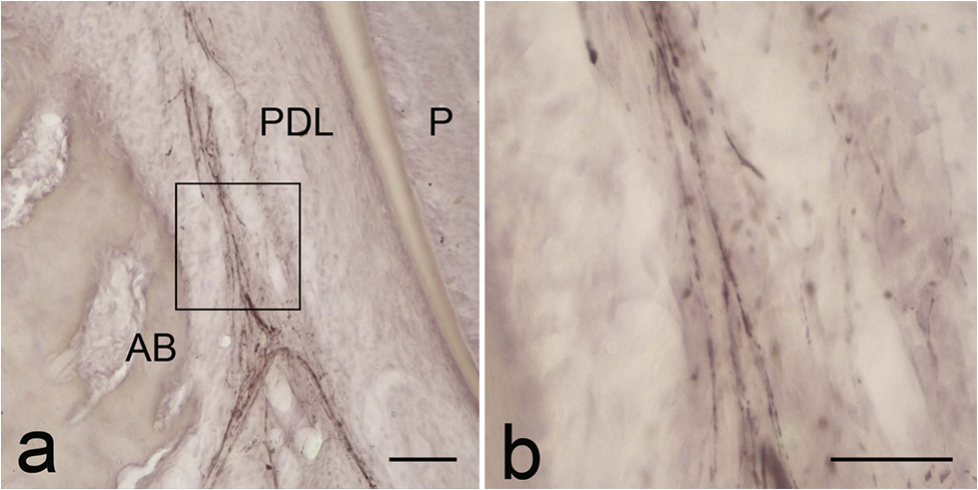
PGP9.5 immunoreactivity in the periodontal ligament of the lower permanent molar of the 5-month-old cat. a: A few nerve fibers are found in the periodontal ligament (PDL). AB; alveolar bone. P: dental pulp b; High-powered image of the boxed area in a. PGP9.5-immunopositive thick nerve fibers run along the periodontal ligament. They are less ramified and do not terminate in the cementum. Scale bars: 100 μm in a and 50 μm in b

In the apical portion of the periodontal ligament of the permanent first molar of the 5-month-old cat, few thin nerve fibers ran along the tooth (Fig 6A and 6B).

**Fig 7:**
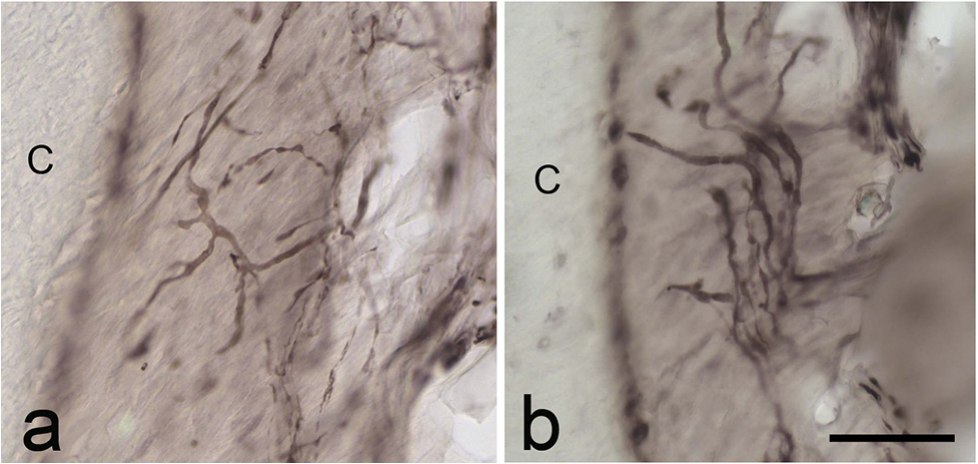
PGP9.5 immunoreactivity in the periodontal ligament of the permanent premolar (a) and molar (b) in a 12-month-old cat. Many thick PGP9.5-immunoreactive thick nerve terminals are observed. They are less expanded. C: cementum Scale bars: 50 μm

In the 12-month-old cat, the periodontal nerve terminals became thicker in the second premolar (Fig 7A), and the first molar (Fig 7B). Thin varicose nerve fibers were also found. The number of periodontal nerve fibers in the permanent second premolar and the first molar of the 12-month-old cat increased compared to that in the 5-month-old cat.

**Fig 8:**
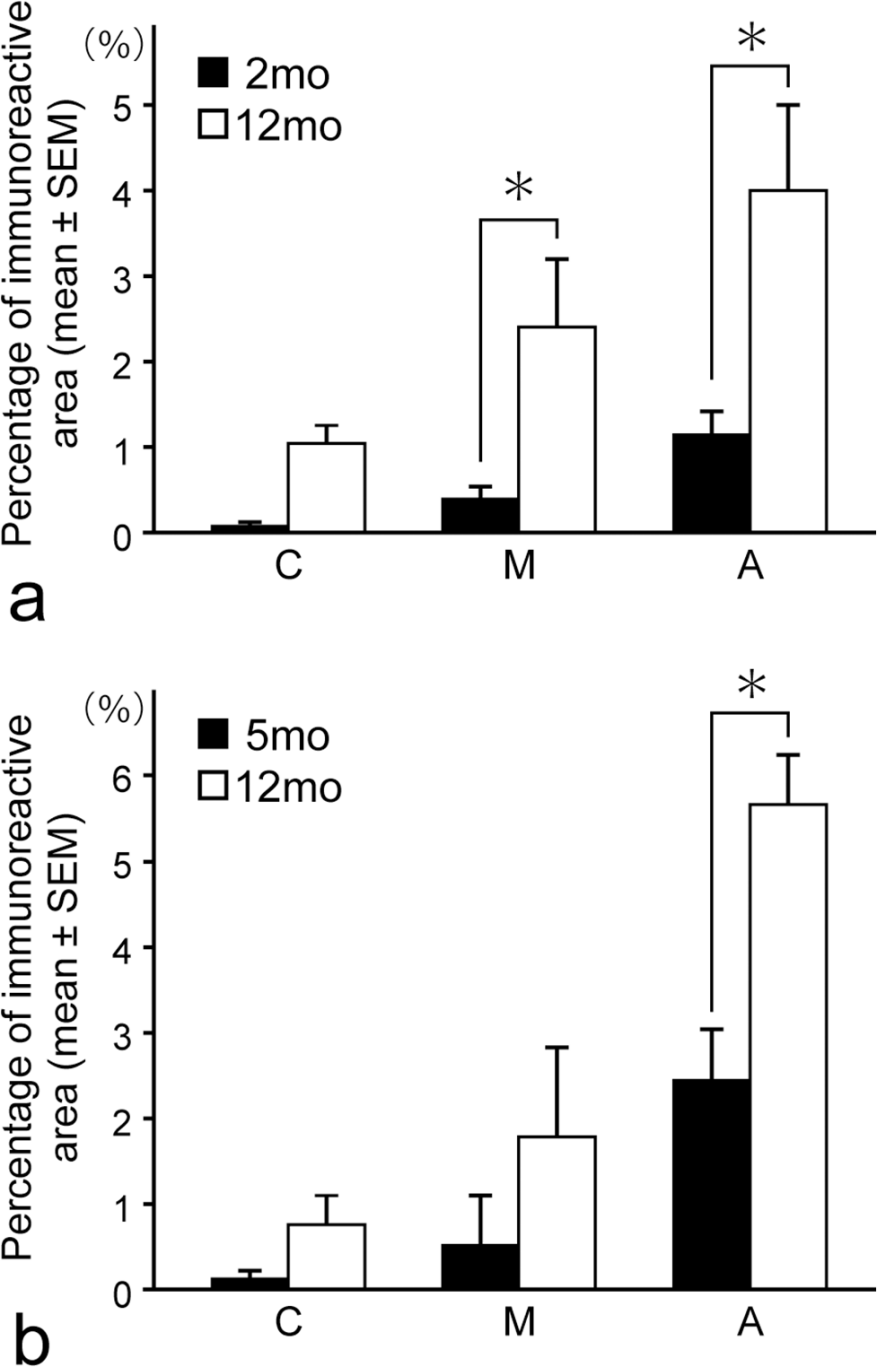
Percentages of PGP9.5-immunoreactive areas on the distal sides of the distal roots of the deciduous second molar in the 2-month-old cats and the permanent second premolar in the 12-month-old cats (a), and the mesial sides of the mesial roots of the permanent first molar in 5- and 12-month-old cats (b) from randomly-selected 4 sections. C: cervical portion, M: middle portion, A: apical portion, as designated in Fig 1.

Fig 8 shows the results of the quantitative analyses. On the distal sides of the distal roots of the deciduous second molar of the 2-month-old cats, approximately 0.02 ± 0.01%, 0.37 ± 0.16%, and 1.12 ± 0.29% (mean ± SEM) of the defined areas were immunoreactive for PGP9.5 in the cervical, middle, and apical portions, respectively. On the distal sides of the permanent second premolar of the 12-month-old cats, approximately 1.03 ± 0.54%, 2.41 ± 0.81%, and 4.07 ± 0.96% of the restricted areas were immunoreactive in the cervical, middle, and apical portions, respectively. The percentages in the middle and apical portions of the permanent second premolar were significantly greater than those of the deciduous second molar.

On the mesial sides of the mesial roots of the permanent first molar in the 5-month-old cats, approximately 0.13 ± 0.08%, 0.85 ± 0.22%, and 2.44 ± 0.58% of the designated areas were immunoreactive for PGP9.5 in the cervical, middle, and apical portions, respectively. On the mesial sides of the mesial roots of the permanent first molar in the 12-month-old cats, approximately 0.72 ± 0.37%, 1.76 ± 1.07%, and 5.66 ± 0.58% (mean ± SEM) of the areas were immunoreactive for PGP9.5 in the cervical, middle, and apical portions, respectively. The apical portion of the mesial sides of the mesial roots of the 12-month-old cats had greater immunoreactive areas for PGP9.5 than that of the 5-month-old cats.

## Discussion

In the present study, we examined morphological changes in the periodontal nerve fibers during transition from deciduous dentition to permanent dentition, and found that, although the periodontal nerve fibers in deciduous teeth was sparsely distributed, the distribution of the periodontal nerve fibers in deciduous teeth was almost identical to that in the succeeding permanent teeth.

Previous studies of periodontal nerve fibers have mostly been performed in rat incisors, where they found that periodontal Ruffini endings of the rat incisor were extensively expanded, and were associated with terminal Schwann cells with kidney-shaped nuclei [1, 2, 3]. In the present study, we showed that the morphology of periodontal nerve fibers in the molars of both deciduous and permanent dentitions was quite different from that of the rat. Our findings are in agreement with previous studies on the morphology of periodontal nerve fibers of the cat molars [5, 7, 8, 9, 10, 11]. Sato *et al*. [12] reported that the nerve terminals of periodontal Ruffini endings in the dog were less expanded, and were associated with the Schwann cell sheath of round-shaped terminal Schwann cells. In the present study, we found that thick nerve fibers were associated with rounded S-100-immunoreactive cells, which are believed to be terminal Schwann cells. Thus, we believe that some periodontal nerve fibers of deciduous teeth are Ruffini endings, although we did not examine their ultrastructural profiles.

Sato *et al*. [16] compared the morphology of periodontal Ruffini endings in the incisors of rodents (guinea pig, hamster, Mongolian gerbil, mouse and squirrel), and found that the innervation pattern of those animals was fundamentally identical to that in the rat incisor [17]. Similar Ruffini endings have been reported in the periodontal ligament of rodent molars [4, 18, 19]. In the present study, we found that periodontal nerve endings, including presumable Ruffini endings, of the cat were less expanded, and were similar to the periodontal nerve endings that have been reported in dogs [12], monkeys [5,13] and humans [6]. We have no exact explanation as to why periodontal nerve endings of the cat are less expanded compared with that of rodents. One possible explanation is a difference in dentition. Rat and other rodents, having extensive ramified periodontal Ruffini endings, are monophyodonts, and animals having less expanded Ruffini endings, such as the dog, cat, monkey and human, are diphyodonts. Periodontal nerve fibers of monophyodont animals do not need to degenerate; thus, nerve endings can expand. It is known that the incisors of rodents are rootless and continuously erupting, which is different from the molars. Many studies [1, 2, 3] have revealed that periodontal Ruffini endings are localized in the alveolar half of lingual periodontal ligament, and rarely in the tooth half of the ligament—the alveolar half is fixed part and tooth half is moving part [20]. Thus, even in the rat incisor, periodontal Ruffini endings do not need to degenerate because continuous eruption. In contrast, in diphyodont animals, periodontal nerve fibers in deciduous dentition have to degenerate due to the resorption of the root of deciduous teeth and eruption of successional permanent teeth, resulting in less-expanded periodontal nerve endings. This explanation is supported by the findings that the periodontal nerve fibers in the crocodile, whose dentition is polyphydonty, are less expanded [21].

It has been previously shown in the postnatal development of rat that the periodontal Ruffini endings mature with completion of occlusion [22, 23], suggesting that the occlusal force is an important environmental factor for maturation of periodontal nerve fibers. This speculation is supported by our previous findings that a reduction of occlusal forces during development causes the delay of development and morphological maturation of periodontal nerve fibers in the rat [15]. In the present study, we found that periodontal nerve fibers in the permanent first molar of the 5-month-old cat are sparsely distributed compared with that of the 12-month-old cat—the percentages of immunoreactive areas in the 12-month-old cat were greater than that in the 5-month-old cat. Thus, we can conclude that, similar to the rat, the occlusal force is an important factor for maturation of periodontal nerve fibers in the cat.

During mixed dentition (i.e., 5-month-old cat), we found many thick nerve fibers having a dot-like appearance in the periodontal ligament just below the resorbing root of deciduous second molar and at the cervical part of the ligament of the permanent premolar. We believe that these nerve fibers were degenerating nerve fibers innervating the periodontal ligament of the resorbing deciduous second molar. In the apical part of permanent second premolar, some thick nerve fibers showed a dot-like appearance, while others did not. We believe that the former innervates the periodontal ligament of the resorbing deciduous second molar and the latter innervates the periodontal ligament of the erupting permanent second premolar.

There is a debate as to whether deciduous teeth and successional teeth are innervated by the same neuron. Electrophysiological analysis revealed that some nerve fibers supplying the deciduous dental pulp are retained to supply its permanent successors in the cat [24], while the shedding of deciduous teeth caused degeneration in the trigeminal brainstem [25]. It has been reported that some trigeminal ganglion neurons innervate more than two tooth pulps in the cat having permanent dentition [8, 26]. As trigeminal ganglion neurons send axons to the periodontal ligament and the tooth pulp, it is easily speculated that some trigeminal ganglion neurons also innervate the periodontal ligament of more than two teeth in permanent and deciduous dentitions. However, it is unknown whether a single neuron in the trigeminal mesencephalic nucleus, another source of periodontal nerve fibers, innervates the periodontal ligament of more than two teeth. If a single trigeminal neuron innervates deciduous and permanent teeth, collateral axons innervating the deciduous teeth should degenerate during the succession while another collateral axons innervating its successor should be retained even though they have the same parent axon.

Clinically, children are less sensitive to stimulation of dentine compared with adults. These clinical observations are supported by the evidence that dental pulps of deciduous teeth are sparsely innervated compared with that of permanent teeth [27, 28]. The present findings also suggest that children may also be less sensitive to stimulation of the periodontal ligament compared to adults, although it is difficult to explain periodontal sensation correctly even in adults.
